# A case report to assess the safety and efficacy of Burosumab, an investigational antibody to FGF23, in a single pediatric patient with Epidermal Nevus Syndrome and associated hypophosphatemic rickets

**DOI:** 10.1016/j.bonr.2022.101605

**Published:** 2022-07-20

**Authors:** Carson Huynh, Andrea Gillis, Jessica Fazendin, Hussein Abdullatif

**Affiliations:** University of Alabama at Birmingham Department of Pediatrics, United States of America; University of Alabama at Birmingham Department of Surgery, United States of America

**Keywords:** Burosumab, FGF23, Hypophosphatemia, Hyperparathyroidism, Epidermal Nevus Syndrome

## Abstract

Epidermal Nevus Syndrome (ENS), also known as Cutaneous Skeletal Hypophosphatemia Syndrome or Linear Sebaceous Nevus Syndrome, is caused by a mosaic somatic mutation of *RAS* (Rat Sarcoma genes) which leads to abnormally elevated levels of fibroblast growth factor 23 (FGF23). FGF23 is a major regulator in phosphate homeostasis. There are multiple disorders, along with Epidermal Nevus Syndrome (ENS), that result in unusually high circulating levels of FGF23. This increase ultimately leads to renal phosphate wasting and reduced levels of 1,25-dihydroxy vitamin D. Across these disorders, the clinical symptoms are similar and often include osteomalacia (hypophosphatemic rickets in children), muscle weakness, fatigue, joint deformities, bone pain, and fractures. Burosumab (KRN23), is an IgG1 monoclonal antibody that binds to the FGF23 receptor and inhibits the activity of FGF23. This leads to an increase in serum phosphate levels. Burosumab emerged as a potential therapy in FGF23 overactivity disorders. Burosumab was successful in the treatment of X-linked hypophosphatemia (XLH) and is now FDA-approved for its treatment. Studies have indicated that Burosumab therapy in subjects with XLH consistently increases and sustains serum phosphorus levels and tubular reabsorption of phosphate without a major impact on urine calcium levels or vitamin D metabolism. We studied the effect of Burosumab treatment in a single pediatric patient with Epidermal Nevus Syndrome. Serum phosphorus rose gradually as we titrated the dose of Burosumab upwards. During treatment, a persistent elevation of parathyroid hormone levels was noted along with a persistent elevation of serum calcium. We presumed the patient had tertiary hyperparathyroidism. However, after the removal of three parathyroid glands, the pathology came back with a single enlarged parathyroid adenoma. Subsequently, his calcium and PTH, and phosphorus levels stabilized while taking only Burosumab.

ClinicalTrials.gov NCT04320316.

## Highlights

• Burosumab was superior to conventional therapy in our subject with ENS.
• The subject had persistent hyperparathyroidism during treatment.
• Hyperparathyroidism corrected with removal of parathyroid adenoma

## 1 Introduction

Epidermal Nevus Syndrome (ENS) is a congenital, multisystem disorder that is characterized by multiple cutaneous epidermal nevi that produce abnormally high levels of fibroblast growth factor 23 (FGF23) (Goyal et al., 2020). Under normal physiologic conditions, FGF23 is primarily produced in the bone and functions as a key regulator of phosphate and vitamin D metabolism (Imel et al., 2019a). FGF23 enters the circulation and functions to reduce the number of sodium-phosphate transporters in the proximal convoluted tubule, thereby suppressing phosphate reabsorption. Additionally, FGF23 has roles in vitamin D metabolism. FGF23 acts to suppress the activity of renal 1α-hydroxylase, leading to decreased levels of active 1,25-dihydroxy vitamin D. This marked decrease in 1,25-dihydroxy vitamin D leads to reduced intestinal absorption of calcium and phosphate (Shimada et al., 2004). Renal phosphate wasting and decreased activity of 1,25-dihydroxy vitamin D leads to clinical manifestations in ENS, especially those of the skeletal system due to bone demineralization. These include osteomalacia (rickets in children), joint deformities, bone pain, and fractures (Sugarman and Reed, 1969; Hosalkar et al., 2003).

The expression of FGF23 is regulated by RAS. In ENS, there is a mosaic somatic mutation of RAS leading to elevated expression of plasma FGF23 (Khadora and Mughal, 2021). Along with ENS, there are several disorders in which FGF23 levels are abnormally elevated. These disorders share common clinical symptoms, especially those skeletal in nature, with ENS due to similar pathophysiology. Since the identification of the role of elevated FGF23 in such disorders, there have been multiple studies analyzing the effects of neutralizing anti-FGF23 receptor antibodies. X-linked hypophosphatemia (XLH), is a disorder that involves a mutation in the Phosphate Regulating Endopeptidase Homolog X-Linked (*PHEX*) gene, leading to an overexpression of FGF23 (Schindeler et al., 2020). Like ENS, this leads to renal phosphate wasting and decreased 1,25-dihydroxy vitamin D, resulting in poor bone mineralization. Conventional therapy consists of phosphate and vitamin D supplementation to treat or to offset the development of osteomalacia and hypophosphatemic rickets (Linglart et al., 2014). More recently, Burosumab, an IgG1 monoclonal antibody towards the FGF23 receptor, has been identified as a potential therapy for XLH (Imel et al., 2019a).

There is consistent clinical evidence that Burosumab is beneficial when used in patients with XLH. In adult clinical trials, Burosumab has been shown to normalize serum phosphorus and improve quality of life (Schindeler et al., 2020). Similar evidence has been found in pediatric clinical trials. An international multicenter trial compared Burosumab to the conventional therapy of calcium and active vitamin D supplementation in pediatric patients ages 1–12 with XLH (Imel et al., 2019b). The study found significant and rapid improvement in serum phosphate levels in the group receiving Burosumab. These patients also showed a statistically significant improvement in the Thacher rickets severity score, improvement in height, and had greater toleration in a distance walked test when compared to the group given conventional treatment. Long-term treatment with Burosumab in pediatric patients with XLH has shown sustained efficacy and similar positive effects (Linglart et al., March 2022).

Given these positive results of Burosumab treatment in patients with XLH, we hypothesize that these results can also be achieved in other FGF23 overactivity disorders, such as ENS. In this study, we examine the results of a 12-month course of Burosumab injections in a 15-year-old male with ENS and associated hypophosphatemic rickets.

Our subject is a 15-year-old male who was diagnosed with ENS at the age of 2 months and was diagnosed with hypophosphatemia at the age of 1 year. At this time, he was started on conventional therapy and continued until the initiation of therapy. The regimen is as follows: calcium carbonate 2000 mg 4 times daily, Phospha 250 neutral oral tablets (8 tablets 4 times daily), and calcitriol 0.5 mg twice daily. His phosphorus level during the last three years before the initiation of Burosumab fluctuated between 0.7 mg/dL and 2.8 mg/dL (normal phosphorus is between 3.5 mg/dL and 5.9 mg/dL). During his lifetime he was diagnosed with multiple pathologic fractures and subsequent orthopedic surgeries. At the time of the initiation of therapy, he was dependent on a motorized wheelchair with significant scoliosis and musculoskeletal pains.

Before the imitation of therapy, his plasma PTH levels were found to be markedly elevated. It is possible that this hyperparathyroidism was secondary due to the prolonged treatment with phosphate and calcitriol. Similar findings are demonstrated in patients with XLH. It is well known that in patients with XLH, prolonged exposures to similar conventional therapy puts patients at risk for developing secondary and tertiary hyperparathyroidism (DeLacey et al., 2019).

## 2 Materials and methods

The patient and his father were informed of the study and were consented to the treatment and protocol plan. The study was approved by our local IRB. Burosumab and the study expenses were covered by a grant from Ultragenyx. At baseline, we asked the patient to stop his current therapy 1–2 weeks before the start of the Burosumab treatment. We obtained baseline levels of PO4, PTH, Calcium, comprehensive metabolic profile, 1,25-dihydroxy vitamin D levels, skeletal survey, DEXA scan, 24-hour urine phosphorus, calcium, and creatinine values as well as FGF-23 levels just before the first dose of Burosumab. The starting dose of Burosumab was 0.3 mg/kg given by subcutaneous injection every 2 weeks. The dose was adjusted upwards every four weeks by increments of 0.1 mg/kg as long as the phosphorus level is below the lower limit of the normal range. By the end of the study, his dose reached 1.4 mg/kg/dose. We checked serum phosphorus 2–3 days before each injection and we repeated the baseline labs at the six- and 12-months visits.

ClinicalTrials.govNCT04320316.

## 3 Results

**Fig. 1:**
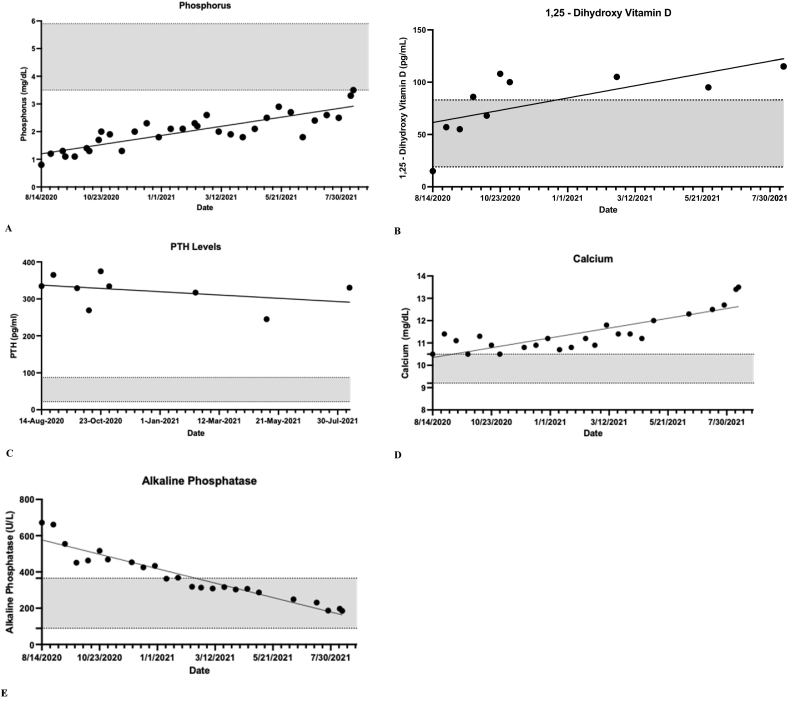
Serum levels of phosphorus, 1,25-hydroxy Vitamin D, parathyroid hormone, calcium, and alkaline phosphatase. Gray boxes indicate normal ranges. A. Serum phosphorus levels for 12-months treatment with Burosumab. B. Serum 1,25-dihydroxy vitamin D levels for 12-months treatment with Burosumab. C. Serum parathyroid hormone levels for 12-months treatment with Burosumab. D. Serum calcium levels for 12-months treatment with Burosumab. E. Serum alkaline phosphatase levels for 12-months treatment with Burosumab.

Throughout the course of the 12-month treatment, phosphorus levels trended upward and eventually normalized at a high of 3.5 mg/dL (Fig. 1A). Likewise, 1,25-dihydroxy vitamin D trended upward rapidly towards a high of 115 pg/mL (Fig. 1B). Serum PTH remained persistently elevated with a subtle downward trend (Fig. 1C). Calcium levels were at the upper normal range throughout the majority of the course and began to uptrend during the later stages of treatment towards a high of 13.5 mg/dL (Fig. 1D). Alkaline phosphatase levels downtrended and normalized throughout treatment and reached a low of 185.2 U/L (Fig. 1E).

With the persistent elevation of calcium and PTH levels, he was referred to endocrine surgery. He underwent a bilateral cervical parathyroid exploration which revealed a single enlarged parathyroid adenoma weighing 1.052 g on final pathology. Post-operatively his calcium level was maintained at 9.5 mg/dL with a PTH level of 35 pg/mL. Two months post-surgery, his only medication was Burosumab and his calcium level was 10.2 mg/dL (normal calcium is between 9.2 mg/dL and 10.5 mg/dL), phosphorus 5.1 mg/dL (normal phosphorus is between 3.5 mg/dL and 5.9 mg/dL), and PTH of 55 pg/mL (normal PTH is between 21.9 pg/mL and 87.6 pg/mL).

Clinically, the patient showed subjective improvement in strength and pain. Because the patient is wheelchair bound, we were not able to perform strength questionnaire surveys and had to accept his subjective reports. The patient reported improvement in his musculoskeletal pain after each injection and this pain returned 2–3 days before the next dose of the Burosumab. He subjectively felt stronger but that was harder to quantitate since he was mostly wheelchair-bound. Other than injection site pain he did not report any other adverse events.

## 4 Discussion

Our study on a single 15-year-old male with ENS and hypophosphatemic rickets showed that Burosumab is effective and superior to the conventional treatment for hypophosphatemic rickets. On conventional therapy, his phosphorus level was frequently below 1 mg/dL. After treatment with Burosumab, our results were similar to studies with patients with X-linked hypophosphatemic rickets who also received treatment. In studies in both adult and pediatric patients with XLH, Burosumab treatment has been associated with elevation and normalization of serum phosphorus levels (Imel et al., 2015).

Additionally, in our study, serum total 1,25-hydroxy vitamin D rapidly increased and remained consistently elevated throughout the duration of treatment. FGF23 is known to reduce renal 1α-hydroxylase activity leading to decreased levels of 1,25-dihydroxy vitamin D. This increase in serum 1,25-dihydroxy vitamin D is expected with Burosumab. Similar rapid elevation in 1,25-dihydroxy vitamin D levels has been described in patients with XLH receiving Burosumab treatment (Imel et al., 2015).

Interestingly, in the course of our study, serum PTH levels remained consistently elevated. At the beginning of the study, serum PTH levels were markedly elevated, possibly due to a secondary hyperparathyroidism due to the length of time on conventional therapy. It is possible that Burosumab further progressed the subject into tertiary hyperparathyroidism, due to the length of period that the subject had high levels of parathyroid hormone. However, given the likelihood of patients with FGF23 overactivity disorders, such as XLH, progressing into tertiary hyperparathyroidism on conventional therapy (DeLacey et al., 2019) it is likely that the subject would have similarly progressed into tertiary hyperparathyroidism if he remained on initial therapy. To our knowledge, there are no reports in the literature of hyperparathyroidism in patients with FGF23 overactivity disorders treated with Burosumab.

Subsequently, the subject underwent a parathyroid scan and evaluation by endocrine surgery. He then underwent surgical removal of an adenomatous parathyroid gland with normalization of both calcium and PTH levels. Two months post-surgery, he is only receiving Burosumab, and his labs showed the highest phosphorus level of 5.1 mg/dL with normal calcium of 10.2 mg/dL and PTH of 55 pg/mL. This prompted us to lower his current dose of Burosumab.

It is likely that this adenomatous parathyroid gland was the main contributor in the progression of the subject's hyperparathyroidism. Additionally, we suggest further investigation should look at patients with established hyperparathyroidism being treated with Burosumab.

Currently, the use of Burosumab is gaining much interest in disorders related to FGF23 overactivity. Such disorders, such as fibrous dysplasia/McCune-Albright syndrome, have similarly shown that Burosumab is superior to conventional treatment (Gladding et al., 2021). To our knowledge, our report is one of the first to study the effects of Burosumab in ENS (Khadora and Mughal, 2021). We hope that this report, in addition to others, will highlight the need for further investigation into the use of Burosumab in ENS and other FGF23 overactivity disorders.

## CRediT authorship contribution statement

**Carson Huynh:** Writing – original draft, Formal analysis, Visualization. **Andrea Gillis:** Writing – review & editing. **Jessica Fazendin:** Writing – review & editing. **Hussein Abdullatif:** Supervision, Conceptualization, Methodology.

## Declaration of competing interest

The authors have no affiliation with any organization with a direct or indirect financial interest in the subject matter discussed in the manuscript.
